# Assessment of GSK1904529A as a promising anti-osteosarcoma agent

**DOI:** 10.18632/oncotarget.17911

**Published:** 2017-05-16

**Authors:** Hao-Dong Fei, Qi Yuan, Li Mao, Feng-Li Chen, Zhao-Hui Cui, Sha Tao, Feng Ji

**Affiliations:** ^1^ Department of Orthopedics, Huai'an First People's Hospital, Nanjing Medical University, Huai'an, China; ^2^ Department of Endocrinology, Huai'an First People's Hospital, Nanjing Medical University, Huai'an, China; ^3^ Clinical Laboratory, Huai'an First People's Hospital, Nanjing Medical University, Huai'an, China

**Keywords:** osteosarcoma, GSK1904529A, IGF1R, proliferation

## Abstract

The insulin growth factor-I receptor (IGF1R) signaling is a key mechanism for osteosarcoma (OS) cell proliferation. GSK1904529A is a novel small molecule IGF1R kinase inhibitor. Its activity against OS cells was tested. In both established OS cell lines (Saos-2 and MG-63) and primary human OS cells, treatment with GSK1904529A (at nM concentrations) significantly inhibited cell proliferation. At the molecular level, GSK1904529A almost completely blocked IGF1R activation in OS cells, and inhibited downstream AKT-ERK activation. IGF1R silence by targeted shRNA also inhibited AKT-ERK activation and Saos-2 cell proliferation. Significantly, GSK1904529A was unable to further inhibit proliferation of IGF1R-silenced Saos-2 cells. *In vivo*, GSK1904529A administration orally inhibited Saos-2 tumor growth in nude mice. Together, these results suggest that targeting IGF1R by GSK1904529A inhibits OS cell growth *in vitro* and *in vivo*.

## INTRODUCTION

Over the past decades, the prognosis of osteosarcoma (OS) has been improved [1–5]. The five-year overall survival of OS has yet reached a plateau [1–5]. The incidence of OS has been rising in the world [6, 7]. Molecule-targeted therapy has drawn significant attentions in recent years. A better understanding of molecular pathology could likely lead to further improvements of OS treatment [8–10].

The insulin growth factor-I (IGF-I)/IGF-I receptor (IGF1R) pathway has been considered as an important contributor for OS tumorigenesis and progression [11–13]. Over-expression and/or hyper-activation of IGF1R tyrosine kinase will lead to constitutive activation of downstream signalings, including the insulin receptor substrate-1 (IRS-1) as well as AKT-ERK cascade [14, 15], which are important for a number cancerous behaviors, including cell proliferation and survival [11–13]. On the other hand, IGF1R inhibition, using genetic or pharmacological methods, could potently inhibit OS cells [11–13]. Thus, IGF1R is a valuable oncotarget protein of human OS.

Recent studies have developed GSK1904529A as a novel and potent small molecule IGF1R kinase inhibitor [16–19]. Preclinical evidences have demonstrated that GSK1904529A, by blocking IGF1R signaling, could possibly inhibit proliferation of several tumor cells [16–19]. To our best knowledge, it potential effect in human OS cells has not been studied thus far. Our results here indicate that targeting IGF1R signaling by GSK1904529A inhibits OS cell growth *in vitro* and *in vivo*. GSK1904529A could possibly have promising translational value for OS treatment.

## RESULTS

### GSK1904529A inhibits Saos-2 cell proliferation

**Figure 1:**
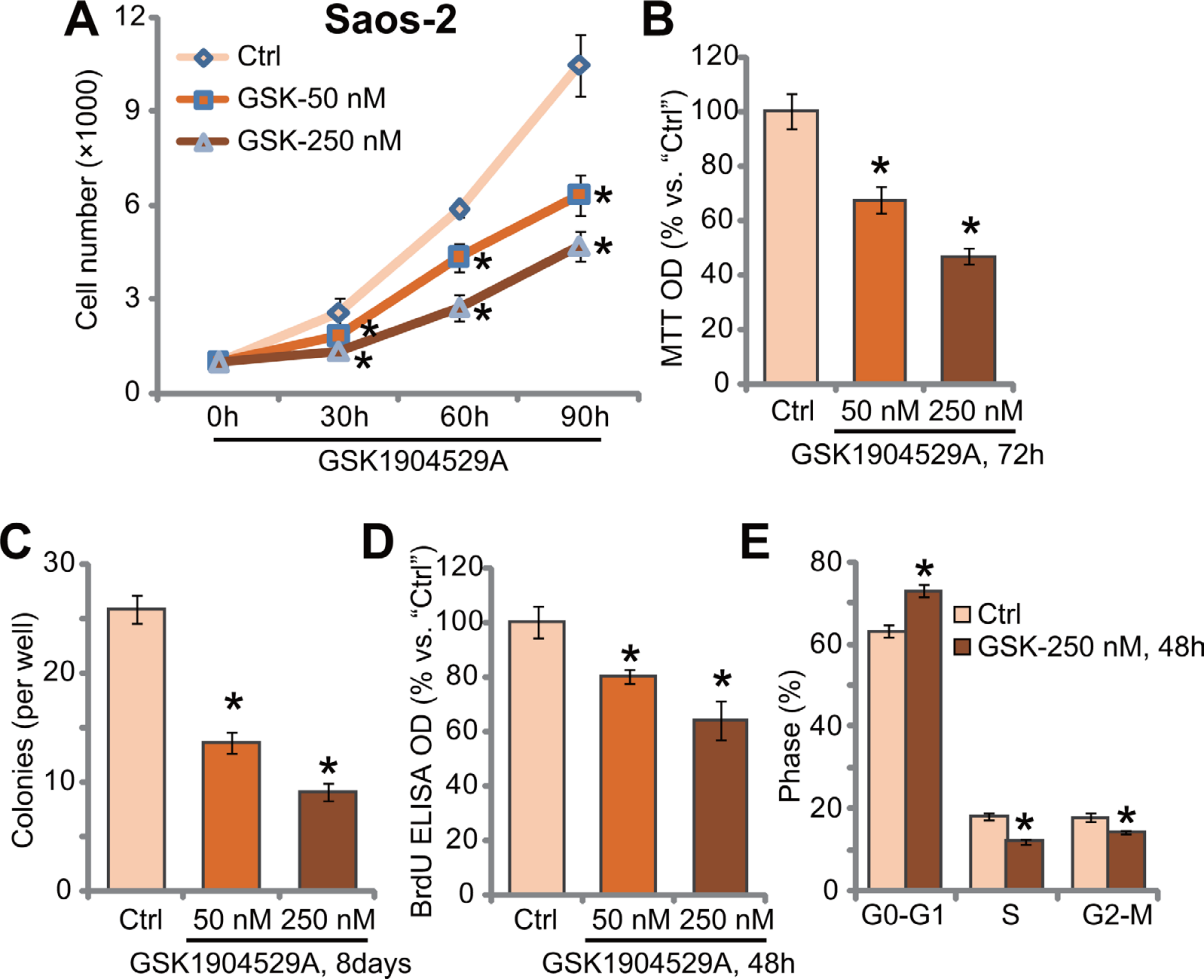
GSK1904529A inhibits Saos-2 cell proliferation Saos-2 cells were either untreated (“Ctrl”, same for all Figures) or treated with GSK1904529A at designated concentration (50/250 nM), cells were further maintained in the conditional medium for indicated time; Listed assays were performed to tested cell proliferation (**A**–**E**). Data were expressed as mean ± SD (*n* = 5). *p < 0.05 *vs*. “Ctrl”. Experiments in this figure were repeated three times, and similar results were obtained.

In order to study the potential effect of GSK1904529A, human OS Saos-2 cells [20, 21] were treated with GSK1904529A. Cell counting assay results in Figure 1A demonstrated that treatment GSK1904529A, at 50/250 nM, significantly inhibited Saos-2 cell proliferation. The number of viable Saos-2 cells was significantly reduced following GSK1904529A (50/250 nM) treatment, at 30-, 60-, and 90-hour durations (Figure 1A). GSK1904529A displayed a concentration-dependent manner in inhibiting Saos-2 cell proliferation (Figure 1A). Treatment with GSK1904529A in Saos-2 cells also dramatically decreased MTT OD (Figure 1B) and number of colonies (Figure 1C). GSK1904529A at 250 nM was again more potent than 50 nM in inhibiting Saos-2 cell proliferation (Figure 1B and 1C).

BrdU incorporation is a characteristic marker of cell proliferation. Results in Figure 1D showed that GSK1904529A treatment dose-dependently inhibited BrdU ELISA OD of Saos-2 cells, again confirming its anti-proliferative activity. Cell cycle analysis results in Figure 1E showed that GSK1904529A (250 nM) treatment induced increase of G0-G1 phase Saos-2 cells, but decrease of S phase and G2-M phase cells. Thus, GSK1904529A likely induced G1-S arrest in Saos-2 cells (Figure 1E). Collectively, these results suggest that GSK1904529A inhibits Saos-2 cell proliferation.

### GSK1904529A is anti-proliferative to MG-63 cells and primary human OS cells

**Figure 2:**
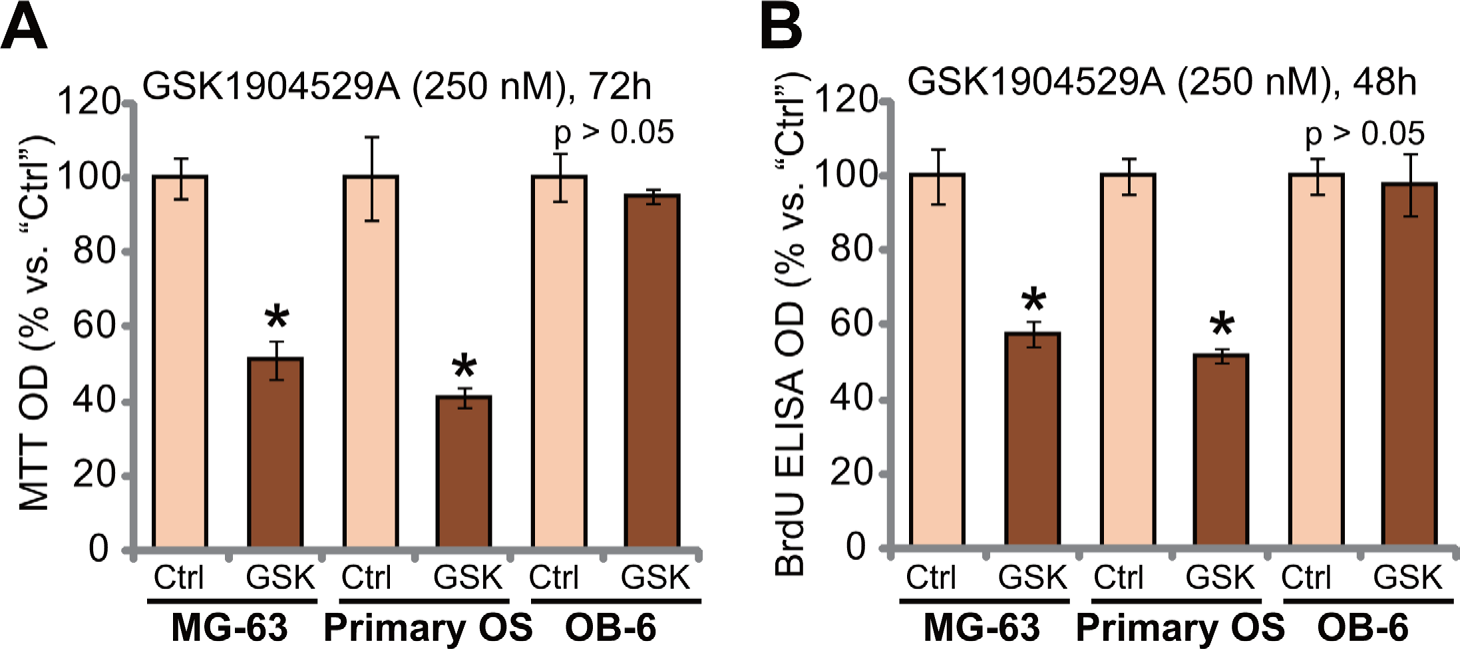
GSK1904529A is anti-proliferative to MG-63 cells and primary human OS cells MG-63 cells, primary human OS cells (“Primary OS”) or human OB-6 osteoblastic cells were treated with/out GSK1904529A (250 nM), cells were maintained in conditional medium for indicated time; MTT assay (**A**) and BrdU ELISA assay (**B**) were performed to test cell proliferation. Data were expressed as mean ± SD (*n* = 5). *p < 0.05 *vs*. “Ctrl”. Experiments in this figure were repeated three times, and similar results were obtained.

We also studied the effect of GSK1904529A in other OS cells. MG-63 is another well-established human OS cell line, which shows IGF1R-dependence [22]. MG-63 cells were treated with GSK1904529A (250 nM). As demonstrated, GSK1904529A treatment significantly decreased MTT OD (Figure 2A) and BrdU ELISA OD (Figure 2B), suggesting proliferation inhibition in MG-63 cells. Next, primary human OS cells were also treated with GSK1904529A (250 nM). Results showed that GSK1904529A also inhibited proliferation of the primary OS cells, evidence by decrease of MTT OD (Figure 2A) and BrdU ELISA OD (Figure 2B). Intriguingly, same GSK1904529A treatment was yet in-effective to OB-6 cells, which are non-cancerous human osteoblastic cells [23, 24]. MTT OD (Figure 2A) and BrdU ELISA OD (Figure 2B) were not significantly changed after GSK1904529A treatment in OB-6 cells (Figure 2A and 2B). Thus, GSK1904529A is selectively anti-proliferative to MG-63 and primary human OS cells.

### GSK1904529A fails to induce OS cell apoptosis

**Figure 3:**
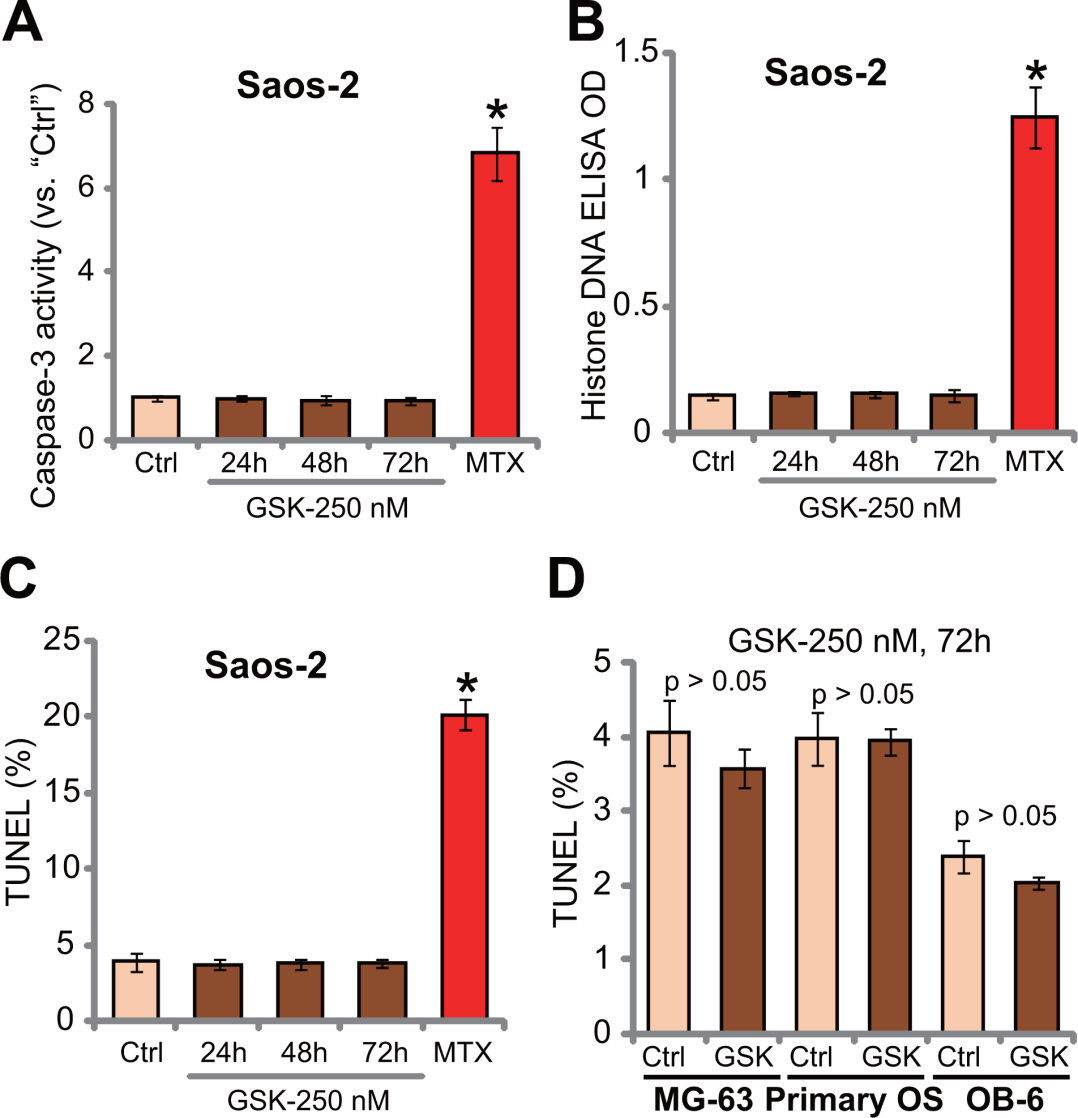
GSK1904529A fails to induce OS cell apoptosis Saos-2 cells (**A**–**C**), MG-63 cells (**D**), primary human OS cells (“Primary OS”) (D) or OB-6 osteoblastic cells (D) were treated with GSK1904529A (“GSK”, 250 nM) or methotrexate (MTX, 10 μM), cells were maintained in conditional medium for indicated time; Cell apoptosis was tested by listed assays (A–D). Data were expressed as mean ± SD (*n* = 5). *p < 0.05 *vs*. “Ctrl”. Experiments in this figure were repeated three times, and similar results were obtained.

The potential activity of GSK1904529A on OS cell apoptosis was tested. In this study, three independent apoptosis assays were applied, including caspase-3 activity assay, histone DNA apoptosis ELISA assay and TUNEL-nuclei staining assay. Results of these apoptosis assays showed that GSK1904529A was unable to significantly induce apoptosis in Saos-2 cells (Figure 3A–3C). Notably, Saos-2 cells were treated with GSK1904529A (250 nM) for 24, 48 and 72 hours, and no significant apoptosis induction was noticed in all the time-points (Figure 3A–3C). On the other hand, methotrexate treatment (10 μM, 48 hours), tested here as a positive control, induced significant apoptosis in Saos-2 cells (Figure 3A–3C). TUNEL assay results in Figure 3D demonstrated that GSK1904529A (250 nM) also failed to activate apoptosis in MG-63 cells and primary human OS cells. No apoptosis was apparently induced in GSK1904529A (250 nM)-treated OB-6 cells (Figure 3D). Collectively, these results suggest that GSK1904529A fails to induce significant apoptosis in OS cells.

### GSK1904529A blocks IGF1R signaling in OS cells

**Figure 4:**
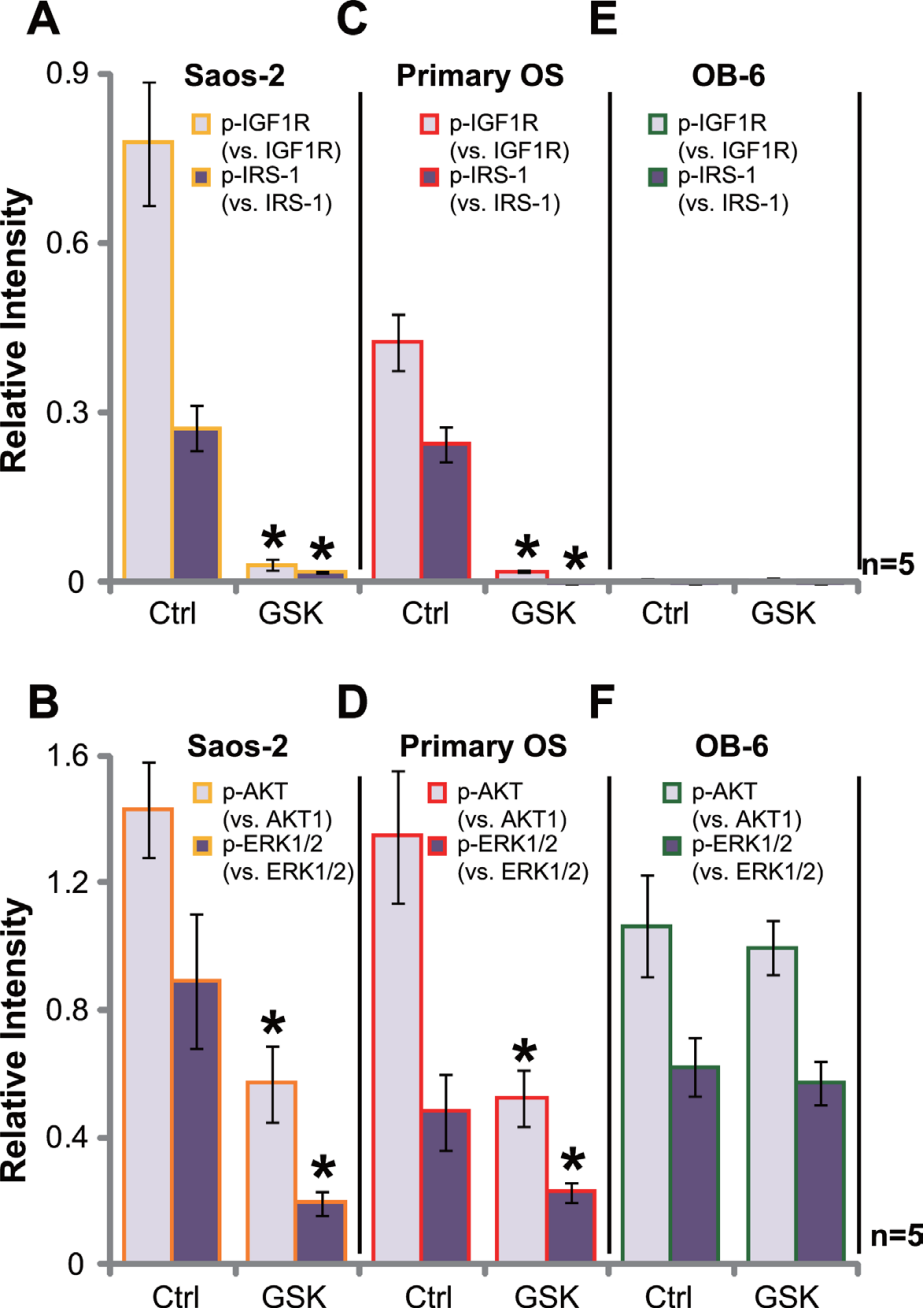
GSK1904529A blocks IGF1R signaling in OS cells Saos-2 cells (**A** and **B**), primary human OS cells (“Primary OS”) (**C** and **D**) or OB-6 osteoblastic cells (**E** and **F**) were treated with/out GSK1904529A (250 nM) for 30 min, expressions of listed proteins were tested by Western blotting assay, data of five repeats were quantified. Data were expressed as mean ± SD (*n* = 5). *p < 0.05 *vs*. “Ctrl”. Experiments in this figure were repeated three times, and similar results were obtained.

GSK1904529A is a novel small-molecule inhibitor of IGF1R tyrosine kinase [16–19]. Its potential effect on IGF1R signaling was tested. Western blotting assay was performed, and quantified results in Figure 4A showed that basal IGF1R activation, tested by phosphorylated (p-)IGF1R and p-IRS-1 (insulin receptor substrate 1), was high in Saos-2 cells, which was almost completely blocked with GSK1904529A (250 nM) treatment (Figure 4A, data were quantified). Consequently, activation of IGF1R downstream signaling, including AKT and ERK, was also largely inhibited by GSK1904529A (Figure 4B, data were quantified). Similar results were also obtained in the primary human OS cells, and GSK1904529A (250 nM) treatment almost blocked IGF1R-IRS-1 activation (Figure 4C), causing AKT and ERK inhibition (Figure 4D). Notably, expression of total above-motioned kinases was unchanged by GSK1904529A. On the other hand, basal activation of IGF1R (p-IGF1R/p-IRS-1) was extremely low in OB-6 osteoblastic cells (Figure 4E), and treatment with GSK1904529A failed to inhibit downstream AKT and ERK activation (Figure 4F). Thus, GSK1904529A apparently blocks IGF1R signaling in OS cells.

### IGF1R knockdown by targeted shRNA abolishes GSK1904529A's activity in Saos-2 cells

**Figure 5:**
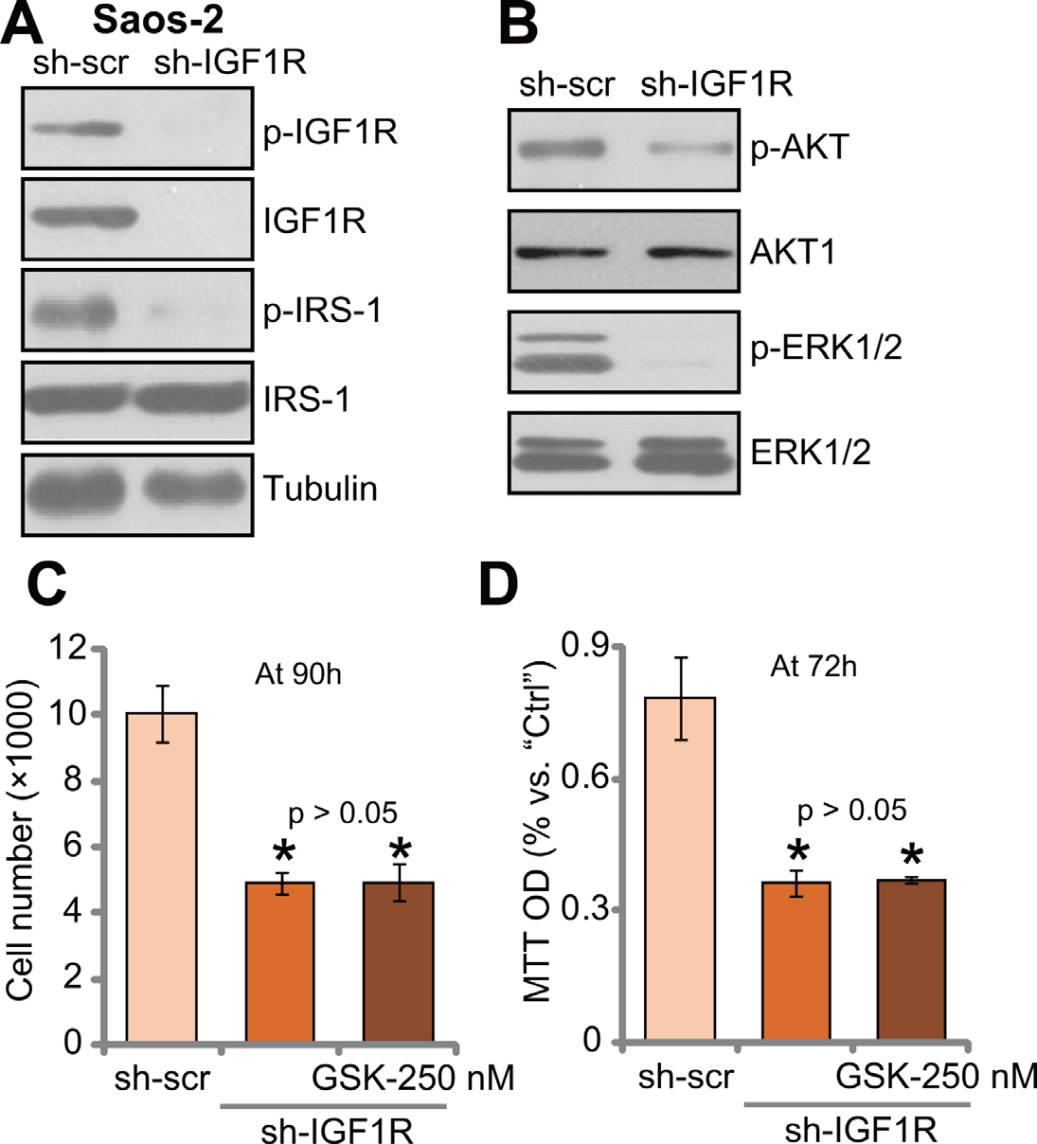
IGF1R knockdown by targeted shRNA abolishes GSK1904529A's activity in Saos-2 cells Exact same amount of stable Saos-2 cells, expressing IGF1R shRNA (“sh-IGF1R”) or scramble control shRNA (“sh-scr”), were subjected to Western blotting assay of listed proteins (**A** and **B**); Cells were also treated with/out GSK1904529A (250 nM) for applied time, and cell proliferation was tested by cell counting (**C**) and MTT assay (**D**). Data were expressed as mean ± SD (*n* = 5). *p < 0.05 *vs*. “sh-scr”. Experiments in this figure were repeated three times, and similar results were obtained.

If IGF1R is the main target of GSK1904529A, it should be in-effective to IGF1R-depleted cells. To test this hypothesis, targeted shRNA was applied to knockdown IGF1R in Saos-2 cells. Western blotting assay results in Figure 5A showed that IGF1R expression was dramatically downregulated in stable Saos-2 cells with IGF1R-shRNA. Consequently, IRS-1 phosphorylation was almost completely blocked (Figure 5A). Further, downstream AKT and ERK activation was also inhibited in IGF1R-silenced Saos-2 cells (Figure 5B). Thus, IGF1R is important for downstream IRS-1, AKT and ERK activation in Saos-2 cells. Significantly, Saos-2 cell proliferation, tested by cell counting (Figure 5C) and MTT assay (Figure 5D), was also inhibited with IGF1R knockdown. Remarkably, treatment with GSK1904529A (250 nM) was unable to further inhibit proliferation of IGF1R-silenced Saos-2 cells (Figure 5C and 5D). Thus, IGF1R knockdown by targeted shRNA likely abolishes GSK1904529A's activity in Saos-2 cells.

### GSK1904529A administration inhibits Saos-2 tumor growth in mice

**Figure 6:**
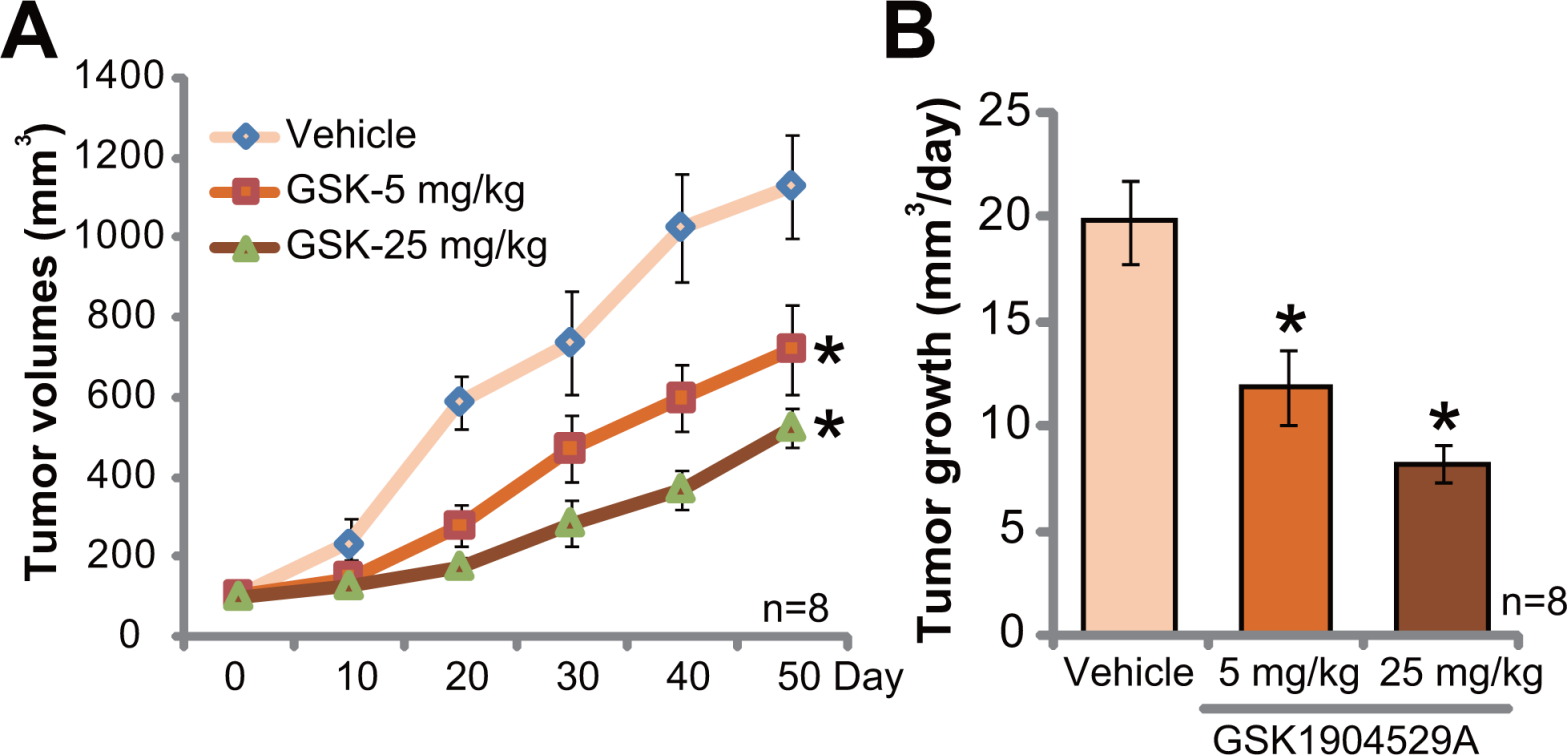
GSK1904529A administration inhibits Saos-2 tumor growth in mice Saos-2 tumor-bearing nude mice were treated with GSK1904529A (5 or 25 mg/kg body weight, gavage, daily for 20 days) or vehicle control (“Vehicle”), tumor volumes (**A**) were monitored every 10 days for 50 days; Daily tumor growth was also calculated (**B**). Data were expressed as mean ± SD (*n* = 8). *p < 0.05 *vs*. “Vehicle” group.

At last, the potential anti-OS activity of GSK1904529A *in vivo* was tested. Saos-2 xenograft tumor model was established. A significant amount of Saos-2 OS cells were *s.c*. injected to the flanks of nude mice to establish xenograft tumors. Mice were then randomly assigned into three groups. GSK1904529A administration regimens were based on previous studies [17–19]. Tumor growth curve results in Figure 6A showed that GSK1904529A oral administration, at 5 and 25 mg/kg daily, significantly suppressed Saos-2 tumor growth in nude mice. GSK1904529A displayed a dose-dependent activity *in vivo*. GSK1904529A at 25 mg/kg was more potent than 5 mg/kg in suppressing Saos-2 tumors (Figure 6A). The results in Figure 6B demonstrated that estimated daily tumor growth, which was calculated by (estimated tumor volume at Day 50 deducting tumor volume at Day 0)/50, was also largely inhibited following GSK1904529A treatment (Figure 6B). Notably, mice body weights of different groups were not significantly different. Neither did we notice any signs of apparent toxicities.

## DISCUSSION

Recent studies implied a pivotal function of IGF1R signaling in cancer progression [13, 25, 26]. Tyrosine kinase activation of IGF1R will recruit IRS-1 and other key adaptor proteins, mediating activation of downstream cascades, including PI3K-AKT-mTOR and MEK-ERK-MAPK signalings [13, 25, 26]. On the other hand, IGF-IR monoclonal antibodies or ATP-competitive IGF-IR inhibitors were tested, which demonstrated promising anti-cancer properties [13, 25, 26].

Here, we showed that GSK1904529A inhibited proliferation of established OS cell lines (Saos-2 and MG-63) and primary human OS cells. GSK1904529A almost completely blocked IGF1R activation, and inhibited downstream AKT and ERK in OS cells. *In vivo*, oral administration of GSK1904529A in nude mice at well-tolerated doses efficiently inhibited Saos-2 tumor growth. Thus, GSK1904529A could be further studied as a promising anti-OS agent.

It has been previously shown that serum IGF level was significantly higher in OS patients than healthy controls, which was associated with a decreased disease-free survival [22]. Meanwhile, IGF-I and IGF-II mRNA upregulation was observed in multiple OS tumor tissues [22]. More importantly, IGR1R, insulin receptor (IR), isoform A of the insulin receptor (IR-A), as well as the IR-A/IGF1R hybrids receptors (HR^A^) were all upregulated in human OS tissues [22]. GSK1904529A, the ATP-competitive IGF-IR inhibitor, could possibly block both IGR1R, IRA and HR^A^ [16–19, 22]. Therefore, GSK1904529A's ability to block both IGF-IR and possible other insulin receptor kinase activity potentially provides a therapeutic advantage over anti-IGF1R monoclonal antibodies.

In the current study, we indicate that IGF1R is required for GSK1904529A's activity against human OS cells. IGF1R silence by targeted shRNA mimicked GSK1904529A ability, and inhibited Saos-2 cell proliferation. More importantly, GSK1904529A was unable to further inhibit Saos-2 cells when IGF1R was silenced. Remarkably, GSK1904529A was also ineffective to human osteoblastic OB-6 cells, where basal IGF1R activation was quite low. GSK1904529A administration *in vivo* was also safe to tested animals. These results suggest a selective response of GSK1904529A against activated IGF1R in OS cells.

## MATERIALS AND METHODS

### Chemicals and reagents

GSK1904529A was obtained from Min-de Biotech (Suzhou, China). Puromycin and methotrexate (MTX) were purchased from Sigma Aldrich (Shanghai, China). Antibodies were purchased from Cell Signaling Technology (Danvers, MA).

### Cell culture

OS cell lines, including Saos-2 and MG-63, as well as human *OB-6* [24] osteoblastic cells, were provided by the Cell Bank of the Chinese Academy of Medical Sciences (Shanghai, China). Cells were maintained in RPMI-1640 medium containing 10% FBS (fetal bovine serum). Cell culture reagents were purchased from Gibco (Suzhou, China).

### Primary human OS cells

Fresh osteoblastoma tissue (from one written-informed patient, male, 12 years old) was minced. Tissues were then subjected to digestion with collagenase I (Sigma). Digestions 2–5 were neutralized, pooled, and filtered. Single cell suspensions of primary human OS cells were re-suspended in the described complete medium [27]. The protocol using primary human tissue was conducted with the principles expressed in the Declaration of Helsinki, and was approved by the institutional review board and Ethics Board of Nanjing Medical University.

### MTT assay

MTT assay was utilized to quantify cell proliferation. Briefly, cells were initially plated in the 96-well plates at 3 × 10^3^ cells per well. After the designated GSK1904529A treatment, MTT dye (Sigma) was added to cells. MTT OD at 490 nm was recorded [28].

### Colony formation assay

After indicated GSK1904529A treatment, Saos-2 cells were plated in 6-well plates at 1 × 10^5^ cells per well. GSK1904529A treatment was renewed every two days for a total of eight days. Afterwards, the remaining Saos-2 cell colonies were fixed, and counted.

### BrdU ELISA assay

Cells with the GSK1904529A treatment were also subjected to BrdU ELISA assay kit (Cell Signaling Tech, Wuxi, China) via the attached protocol [29]. BrdU ELISA OD at 405 nm was recorded to quantify cell proliferation.

### Cell cycle analysis

After the applied GSK1904529A treatment, OS cells were initially fixed by 70% ethanol. Cells were then stained with propidium iodide (PI) and subjected to FACS analysis on a Beckman Coulter flow cytometer (Suzhou, China). Cell cycle distribution was recorded.

### TUNEL assay of cell apoptosis

TUNEL positive staining in cell nuclei is a characteristic marker of cell apoptosis. Cells with applied GSK1904529A treatment were subjected to TUNEL assay kit (Cell Signaling Tech) according to the attached protocol [30]. The ratio of TUNEL positive nuclei (*vs*. total number of nuclei) was recorded under a fluorescent microscope (Leica, Shanghai, China), summarizing at least 200 cells per condition in five independent experiments.

### Histone DNA apoptosis ELISA assay

As described [31–33], Histone DNA enzyme-linked immunosorbent assay (ELISA) kit was applied to test DNA fragmentation, via the commercial available photometric sandwich immunoassay of cytoplasmic histone-associated DNA fragments (Roche, Shanghai, China).

### Caspase-3 activity assay

Following the applied GSK1904529A treatment, Saos-2 cells were subjected to the Apo-ONE homogeneous caspase-3 activity kit (Promega, Shanghai, China), which determines the caspase-3 substrate via Rhodamine 110 fluorescence. Rhodamine 110 fluorescence intensity at 500 nm was recorded as the caspase-3 activity.

### Western blotting assay

Protein lysates (30 μg per lane) were separated by the SDS-PAGE gels, and were transferred to PVDF (polyvinylidene difluoride) membranes. Afterwards, the blots were blocked, and incubated with designated primary and secondary antibodies. Enhanced chemiluminescence (ELC) reagents (Pierce, Shanghai, China) were applied to visualize the targeted protein band/s [32–34].

### IGF1R shRNA

The lentiviral shRNA against human IGF1R was purchased from Santa Cruz Biotech (Wuxi, China), which was directly added to cultured OS cells (10 μL/mL, per well) for 24 hours. Puromycin (2.5 μg/mL, Sigma) was applied to select stable cells for 8–10 days. IGF1R silence was confirmed by Western blotting assay. Control cells were infected with lentiviral scramble control shRNA (Santa Cruz Biotech).

### Tumor xenograft assay

Exponentially growing Saos-2 cells (5 million cells per mouse) were inoculated *s.c*. to the right flank of female nude mice (8- to 9-wk-old, 17.2–18.2 g). When the tumors reached 100 mm^3^ in size, mice were randomized assigned into three groups. Mice were treated orally with the vehicle or 5/25 mg/kg body weight of GSK1904529A, once daily for 20 consecutive days. Tumor volumes were recorded every 10 days. Tumor volume was calculated by the following formula: (length × width^2^)/2.

### Statistical analysis

The quantitative data presented in this study were mean ± standard deviation (SD). Statistical difference was tested via one-way ANOVA with post hoc Bonferroni test.

## CONCLUSIONS

In summary, the results of this preclinical study imply that targeting IGF1R signaling by GSK1904529A inhibits OS cell growth *in vitro* and *in vivo*. GSK1904529A may have significant translational value for human OS treatment.
